# The Hospital System of Sweden

**Published:** 1890-10

**Authors:** 


BUFFALO MEDICAL AND SURGICAL JOURNAL

A MONTHLY REVIEW OF MEDICINE AND SURGERY.

*EDITORS:*

THOMAS LOTHROP, M. D. - - WM. WARREN POTTER, M. D.

All communications, whether of a literary or business character, should be addressed to the editors : 284 FRANKLIN STREET, BUFFALO, N. Y.

VOL. XXX. OCTOBER, 1890. No. 3.

### Editorial.

### THE HOSPITAL SYSTEM OF SWEDEN.

DR. G. BOLLING'S report on the state of the hospital system of Sweden, in 1888, published in *Hygiea* for July, gives a comprehensive view of a subject always interesting to the medical profession, and reflects no small credit on the indefatigable and unselfish workers in this branch of science, while it gives evidence of the enlightened care bestowed upon the art of healing by those having in their keeping the welfare and happiness of their country. Omitting from the report before us details consisting mainly of accounts of new institutions, additions to those already existing, cost of building, keeping, etc., matters of purely local interest, we limit our attention to such points as may be of special interest in studying the general progress of medical science.

The Swedish physician, after his severe training and final examination, though not strictly speaking a public official, except he be in the army or navy, and, therefore, free to pursue his profession in the same manner as our own doctors do, seems to be less subject to the caprice of the public as regards his practice, the well-known high standard of his school being taken by the intelligent masses as the guarantee of his qualification.

In cities the practitioner is generally paid as with us, though, in many instances the family physician receives a stipulated sum by the year, whether his services be required or not.

Public hospitals are known as *Lazarettes*, *Sjuk-hus*, *i. e.*, sick houses, or *Kur-hus*, the last, by the way, being wholly devoted to syphilitic patients ; but whatever the original etymological difference may have been, at present there is none but in name; they may, therefore, be all designated as hospitals.

Some of these institutions are built by, and in part or wholly maintained by private means, but all are under the constituted medical department of the realm, and all are subject to government control, either general or provincial. Another feature in the arrangement for the care of the sick, is the so-called sjuk-stugor (sick cottages). These are placed here and there in some of the remoter and more sparsely settled parts of the country, built and maintained at the expense of the Province, thus enabling the provincial physician to give his scattered patients regular hospital care when necessary.

The physician appointed to the care of a hospital, as well as the provincial doctor, whose duty calls him to all within his district free of charge, though he generally receives voluntary fees from those able to pay, is salaried from public funds and is always a man of ripe experience, a man whose ten years or more of university study, subsequent hospital training and foreign travel have eminently fitted him for his duties as a learned exponent of the mysteries of his profession, and earned for him the confidence of the public as a careful and intelligent practitioner. According to Dr. Bolling's report, the number of institutions for the care of the sick, public and private, was, in 1888, about 130; the number of beds in the public hospitals of the Provinces was 4,401, in civil institutions 2,676; thus, in all, during the year, 7,077 places for patients, or one place for about 671 inhabitants of the country. In all, during the year, 31,645 patients in this way received medical attention. The aggregate cost of maintaining these institutions was about $360,000.

After a tabulated statement of the diseases treated and operations performed, the report closes with a synoptical history of twenty-one remarkable surgical operations, a few of which, of more than ordinary interest, will be found among our translations in a future number of the JOURNAL.

In looking over the present medical literature of the Scandinavian nations, we are struck with the minute attention paid to accuracy of scientific detail and apparent honesty of purpose to state things just as they are, or were, even when error of judgment or unforeseen causes have led to adverse results. Again, we find that capital operations and great results are not confined to the largest cities with their supposed advantages, (true the greater number of cases are treated in the largest and best appointed hospitals,) but some of the most instructive reports of cases, both medical and surgical, are by resident physicians of remote cities and towns, the population of which, in our country, would not entitle them to the name of villages. Great attention is also paid to organic chemistry and hygiene, the chemistry of food products and their physiological or pathological values ; in short, there seems to be no subject connected with the sickness or health of man that is not scientifically and broadly treated in their investigations. When we take into consideration the fact that all the Scandinavian nations together, even including Finland, (now a Russian principality, but Swedish still in feeling and culture,) do not possess a population equal to the combined numbers of New York and Pennsylvania, we must acknowledge that they deserve to rank among the foremost in the advancement of medical science, if in addition to their paucity of numbers we compare their financial resources with the above mentioned States, still greater credit must be awarded their endeavors ; one advantage is theirs, however, in their institutions for the advancement of science, they are practically in unison, and factional strifes, mischievous isms and quackeries, which hamper the honest searcher after truth in more populous and advantageously situated countries, do not so readily take root where intelligence is general and where little is to be gained by greedy charlatans. We would not, however, be understood as claiming that quackery and irregular practice is unknown, but in Sweden, at least, they can never have legal sanction, nor do they seem to be countenanced as here by any considerable part of the people.

